# Population Dynamics of *Aedes aegypti* and Dengue as Influenced by Weather and Human Behavior in San Juan, Puerto Rico

**DOI:** 10.1371/journal.pntd.0001378

**Published:** 2011-12-20

**Authors:** Roberto Barrera, Manuel Amador, Andrew J. MacKay

**Affiliations:** Entomology and Ecology Activity, Dengue Branch, Centers for Disease Control and Prevention, Calle Cañada, San Juan, Puerto Rico; USAMRIID, United States of America

## Abstract

Previous studies on the influence of weather on *Aedes aegypti* dynamics in Puerto Rico suggested that rainfall was a significant driver of immature mosquito populations and dengue incidence, but mostly in the drier areas of the island. We conducted a longitudinal study of *Ae. aegypti* in two neighborhoods of the metropolitan area of San Juan city, Puerto Rico where rainfall is more uniformly distributed throughout the year. We assessed the impacts of rainfall, temperature, and human activities on the temporal dynamics of adult *Ae. aegypti* and oviposition. Changes in adult mosquitoes were monitored with BG-Sentinel traps and oviposition activity with CDC enhanced ovitraps. Pupal surveys were conducted during the drier and wetter parts of the year in both neighborhoods to determine the contribution of humans and rains to mosquito production. Mosquito dynamics in each neighborhood was compared with dengue incidence in their respective municipalities during the study. Our results showed that: 1. Most pupae were produced in containers managed by people, which explains the prevalence of adult mosquitoes at times when rainfall was scant; 2. Water meters were documented for the first time as productive habitats for *Ae. aegypti*; 3. Even though Puerto Rico has a reliable supply of tap water and an active tire recycling program, water storage containers and discarded tires were important mosquito producers; 4. Peaks in mosquito density preceded maximum dengue incidence; and 5. *Ae. aegypti* dynamics were driven by weather and human activity and oviposition was significantly correlated with dengue incidence.

## Author Summary

Previous studies on the influence of weather on *Aedes aegypti* in Puerto Rico suggested that rainfall influenced mosquito populations and dengue incidence in the drier areas of the island. We studied temporal changes in *Ae. aegypti* in areas where rainfall is more uniformly distributed throughout the year. Changes in adult mosquitoes were monitored with BG-Sentinel traps and oviposition activity with CDC enhanced ovitraps. We also counted the number of mosquito pupae in containers with water during the drier and wetter parts of the year to determine the contribution of humans and rainfall to mosquito production. Mosquito dynamics was compared with dengue incidence in the municipalities investigated in the study (November 2007–December 2008). We found that the population of *Ae. aegypti* was driven by weather and human activities, and peaks in mosquito density preceded maximum dengue incidence during the rainy season. Even though Puerto Rico has a reliable supply of tap water and an active tire recycling program, water storage containers (e.g., 5-gal pails, drums) and discarded tires were important mosquito producers. We also documented for the first time that water meters are important producers of *Ae. aegypti*. This longitudinal study contributes to a better understanding of the complex dynamics of weather, human behavior, mosquito vectors, and dengue virus transmission in an endemic country.

## Introduction

There are three main patterns of dengue virus transmission that merit a better understanding of the involvement of *Aedes aegypti*: 1- Major dengue epidemics occur every few years [1], [2]; 2- Dengue viruses have become endemic or hyperendemic (co-circulation of two or more serotypes) in many countries [3], [4], and 3- There is intra-annual, seasonal dengue transmission with peak incidence during the second half of the year in the northern hemisphere and during the first half of the year in the southern hemisphere, each one associated with elevated temperature and precipitation [5]–[7].

There is a lack of long-term, longitudinal studies on the temporal dynamics of *Ae. aegypti* that would, otherwise, allow us to understand whether mosquito outbreaks are responsible for inter-annual dengue epidemics. It is common to observe relatively large densities of *Ae. aegypti* that do not result in major outbreaks, particularly after major epidemics [8], suggesting that other factors such as temporal changes in population immunity or the introduction of new serotypes can significantly influence inter-annual epidemic patterns [9]–[11]. Climate variability, particularly El Niño Southern Oscillation (ENSO) teleconnections with local weather, has been associated with inter-annual dengue epidemics [2], although it would seem that the relationship is complex, non-linear, and perhaps non-stationary [1], [12], [13]. Amarakoon et al. [6] found significant effects of temperature on dengue epidemics in the Caribbean, particularly one year after the onset of an ENSO event.

Because dengue viruses are transmitted by the bite of infected mosquitoes, dengue virus endemicity or hyperendemicity requires the existence of sufficient vectors to produce uninterrupted transmission in spite of adverse, seasonal weather conditions (e.g., lack of rain), such as that observed in urban areas with long dry seasons [14]. Recurrent virus introductions facilitate dengue endemicity. In dengue endemic/hyperendemic countries, dengue viruses are disseminated among regions so that virus re-introductions are frequent and do not depend solely on virus import [4], [15]. There is evidence showing that even in relatively small countries such as Puerto Rico, some dengue genotypes can circulate uninterruptedly for prolonged periods of time [16]. Vertical transmission of dengue virus in *Ae. aegypti* could play a role in the maintenance of endemicity but more evidence is required to understand its role in nature [17]. Perhaps, the single, most important factor determining dengue endemicity is the habit of people of adding water to containers, which can be for drinking, cooking or bathing (water-storage) and for other purposes, such as ornamentation (fountains), watering plants, etc. Production of *Ae. aegypti* in those containers can be so important as to trigger dengue outbreaks during the dry season [18]. Additionally, the existence of cryptic containers with water producing large numbers of *Ae. aegypti* has been more frequently reported [19]–[22], and in at least one occasion, those recondite containers have been linked to local dengue virus transmission [23]. Some cryptic containers, such as septic tanks in Puerto Rico, can produce *Ae. aegypti* throughout the year [24].

There is evidence showing that the intra-annual cycle of dengue transmission is driven by weather and mosquitoes [7]–[9], [25]–[29]. However, there seems to be spatial variability in the temporal dynamics of *Ae. aegypti*. For example, detailed studies of the temporal change in adult and immature stages of *Ae. aegypti* in a temple compound in Bangkok, Thailand in 1966 failed to show an association between weather, mosquitoes, and dengue incidence [30], [31]. The lack of seasonal fluctuation in the *Ae. aegypti* population was explained by the prevalence of containers that were manually filled with water by people, such as water-storage jars and ant-traps. Assuming that there were enough mosquitoes throughout the year, dengue incidence could have increased due to temperature-induced rises in biting rates, and shortened mosquito gonotrophic cycles and virus extrinsic incubations periods that can significantly increase vectorial capacity [32], [33]. On the other hand, entomological surveys conducted in five places in Bangkok in 1962 showed a sharp increase in the number of *Ae. aegypti* at the beginning of the rainy season, followed by a peak in cases of dengue hemorrhagic fever two months later [34]. Similar observations were reported for Koh Samui Island, Thailand [35].

Contrasting observations on the dynamics of *Ae. aegypti* have also been reported in Puerto Rico. Moore et al. [36] found positive correlations between rainfall, immature mosquito populations (Breteau Index), and dengue, and that the relationship between rainfall and mosquitoes was more marked in the drier, southern parts of the island. The authors explained that most larval habitats of *Ae. aegypti* occurred outdoors and were filled at least partly by rain; a result that agrees with more recent surveys [37]. In contrast, Scott et al. [38] did not find any significant associations between rainfall or temperature and female adult *Ae. aegypti* in the wetter, northern San Juan city area. Given that intra-annual dengue transmission is sharply seasonal in Puerto Rico, with maximum transmission during the hot and rainy seasons [39], [40], we decided to investigate the role of weather and human influence on the temporal dynamics of *Ae. aegypti* and dengue in the San Juan metropolitan area. Efficient *Ae. aegypti* adult trap devices (BG-Sentinel) and CDC enhanced ovitraps were used to monitor the mosquito populations. Our observations were structured to minimize temporal and spatial dependence, which tend to undermine p-values in statistical analyses [41]. Also, weather and dengue variables were designed to represent direct mechanistic relationships with the number of mosquitoes. Our results evidenced significant effects of rainfall, temperature, and human behavior on the temporal dynamics of *Ae. aegypti* and dengue in northern Puerto Rico.

## Materials and Methods

### Study sites

**Figure 1:**
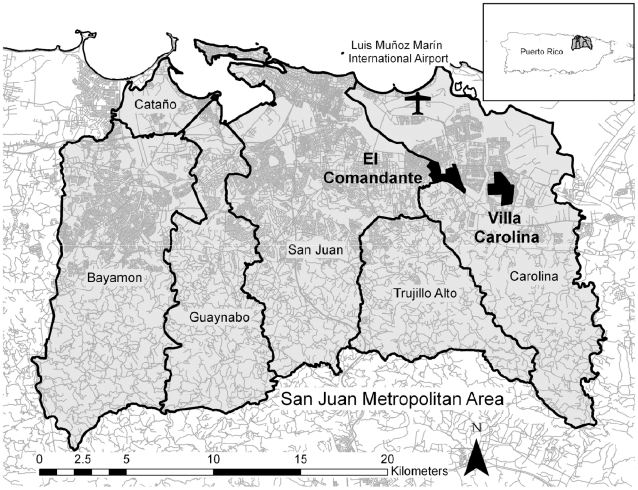
Map of the study areas. The map shows the municipalities of San Juan city, Puerto Rico and the location of the airport in relation to the two neighborhoods investigated. Each neighborhood is composed of two adjacent census tracts.

The study was carried out in two separate neighborhoods, each consisting of two contiguous US census tracts of the Metro Area of San Juan, Puerto Rico (Figure 1). One pair of bordering census tracts was located in urbanization “Villa Carolina”, Carolina municipality (VC; 9240 persons and 1996 buildings; 18°23′52″N 65°57′26″W; US Census 2000).We studied a second pair of adjacent census tracts approximately 3 km from VC: urbanization “Extension El Comandante” in Carolina municipality and urbanization “El Comandante” in San Juan municipality (EC; 6951 persons and 1979 buildings; 18°24′02″N 65°59′30″W; US Census 2000). We will be referring to these latter two census tracts as “El Comandante” in most of the report.

Carolina Municipality has a spatial, insecticide spraying program (truck-mounted Ultra Low Volume equipment) that is active throughout the year, whereas the San Juan Municipality uses a similar insecticide spraying technique but mostly around notified cases of dengue. Thus, both census tracts in VC were subjected to frequent ULV insecticide treatments, whereas in EC only the census tract that belongs to the Carolina Municipality may have been regularly sprayed. Establishing the frequency and coverage of insecticide spraying was attempted but unsuccessful.

### Weather variables

**Figure 2:**
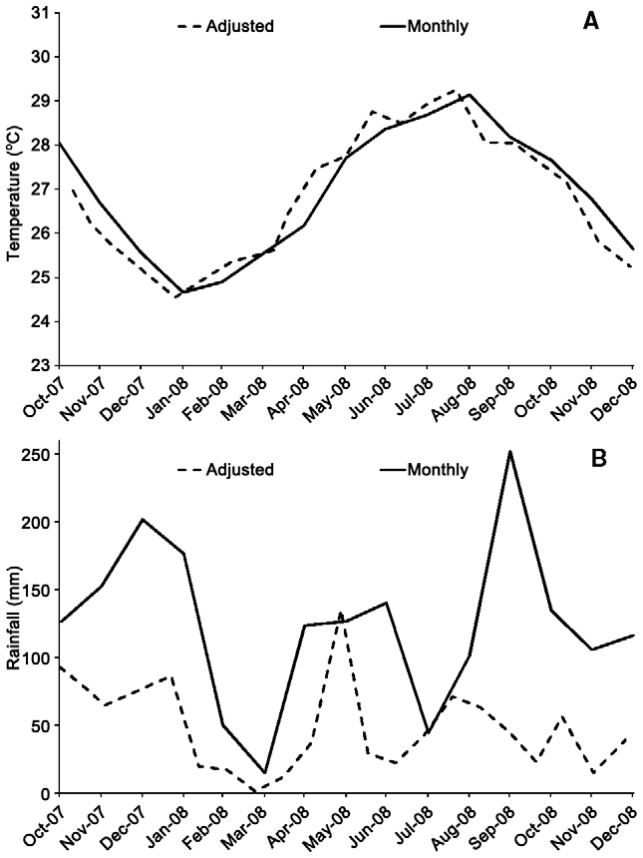
Weather variables at Muñoz-Marin International Airport in 2008, San Juan, Puerto Rico. Panel A shows mean monthly temperature and adjusted temperature. Adjusted temperature is the average of daily mean temperature for 21 days before mosquito sampling. Panel B shows monthly rainfall and adjusted rainfall. Adjusted rainfall is the accumulated rainfall during the third and second weeks before each mosquito sampling, which was conducted every three weeks.

Total annual rainfall and mean daily temperature were 1,388 mm and 27.0°C, respectively at the nearby (4–7 km) Muñoz-Marin International Airport in 2008. Rainfall in the San Juan area occurs year round, with a short, relatively dry season (<100 mm/month) between January and March and two rain maxima around April-May and September-October (Figure 2). Air temperature reached minimum and maximum values in January and August, respectively (Figure 2).

We computed two derived weather variables: adjusted rainfall (ADJRAIN) and temperature (ADJTEMP). Adjusted rainfall was set to be the accumulated rainfall during the third and second weeks before each mosquito sampling date (every three weeks) and adjusted temperature was the average of mean daily temperature for the 21 days before each mosquito sampling date. We did not include rainfall during the week preceding mosquito sampling because the immature cycle of *Ae. aegypti* takes at least seven days. Adjusted temperature was the average of the previous three weeks because this variable could have directly influenced the number of adults present at the time of sampling. It can be observed that adjusted temperature was similar to the average monthly temperature, but adjusted rainfall markedly differed from monthly rainfall (Figure 2).

### Adult *Ae. aegypti* mosquitoes

*Aedes aegypti* adults were concurrently captured in each neighborhood using 40 lured BG-Sentinel mosquito traps (Biogents, Regensburg, Germany) from November 2007 to December 2008 (20 sampling dates each; total 6,107 samples). Each trap was operated for four consecutive days every three weeks. We assumed that the maximum longevity of *Ae. aegypti* was three weeks, so adult mosquitoes captured in consecutive samplings dates should have been born between collection dates, thus minimizing generational overlap and temporal auto-correlations. Collection bags were replaced every day and batteries were replaced after two days of operation. Traps were uniformly distributed across each neighborhood, resulting in inter-trap average distances of 132 m in EC and 137 m in VC to minimize spatial dependence and trap interactions. We calculated the average number of female *Ae. aegypti* captured per trap per day as a measure of relative abundance. We used a Geographical Information System (GIS; ArcView 9.2, Esri, Redlands, CA) to facilitate trap placement. The GIS had the following geo-spatial layers: polygons showing house boundaries (Municipal Tax Revenue Agency, Puerto Rico) and census tracts, digitized lines representing streets, and derived house centroids representing trap locations.

### Ovitrap sampling

In order to compare the number of eggs collected in standard CDC ovitraps [42] with the number of female adults of *Ae. aegypti* in BG traps, and to evaluate relationships with weather variables and dengue, we placed a pair of ovitraps (100% hay infusion or 10% dilution) across the street from the location of each BG trap in both neighborhoods (40 pairs of ovitraps per community). The number of eggs per ovitrap per day for four consecutive days was monitored every three weeks from May to December, 2008 on the same dates as the BGtrap sampling scheme. Eggs were counted under a stereoscope microscope. Given the low prevalence of *Ae. mediovittatus* in the study areas (0.2–0.6% of all container *Aedes* spp.; Table 1), most of the collected eggs should have been *Ae. aegypti*. We averaged the number of eggs in both ovitraps over the four days of collection as a measure of oviposition activity (eggs/ovitrap/day) [43].

**Table 1:** Composition and abundance of adult mosquito species captured in BG traps.

Mosquito species	Villa Carolina(n = 3,048 trap × days)	El Comandante(n = 3,059 trap × days)
*Aedes (Stegomyia) aegypti* (L.) (females)	11,690	14,589
*Ae. (Stg.) aegypti* (L.) (males)	6,534	12,505
*Ae. (Gymnometopa) mediovittatus* (Coquillett)	32	162
*Ae. (Ochlerotatus) taeniorhynchus* (Wiedemann)	25	4
*Ae. (Och.) tortilis* (Theobald)	14	30
*Anopheles (Anopheles) grabhamii* Theobald	28	7
*Anopheles (Ano.) vestitipennis* Dyar & Knab	1	3
*Culex (Culex) bahamensis* Dyar & Knab	7	7
*Cx. (Cux.) chidesteri* Dyar	5	9
*Cx. (Cux.) habilitator* Dyar & Knab	81	214
*Cx. (Cux.) janitor* Theobald	28	37
*Cx. (Cux) nigripalpus* Theobald	52	103
*Cx. (Cux.) quinquefasciatus* Say	11,275	76,425
*Cx. (Melanoconion) atratus* Theobald	149	19
*Cx. (Mel.) iolambdis* Dyar	21	48
*Cx. (Mel.) taeniopus* Dyar & Knab	92	230
*Cx. (Micraedes) antillummagnorum* Dyar	7	1
*Mansonia (Mansonia) flaveola* (Coquillett)	1	0
*Psorophora (Grabhamia) jamaicensis* (Theobald)	0	2
*Uranotaenia (Uranotaenia) cooki* Root	6	0
*Ur. (Ura.) lowii* Theobald	5	2

#### *Aedes aegypti* pupal surveys

To determine the types of containers producing *Ae. aegypti* pupae during the relatively dry season, we conducted simultaneous pupal surveys in VC and EC (15 January–2 February, 2008). The locations of all houses in each study area were incorporated as a point layer in the GIS and used to randomly select houses to be sampled (156 and 152 houses in EC and VC, respectively).We repeated the pupal surveys in both neighborhoods during the beginning of the rainy season (16–25 June, 2008) and increased the sample size to 225 and 235 houses in EC and VC, respectively. The number of houses sampled resulted from a compromise between our capacity: 1- to conduct simultaneous pupal assessments in both communities in a short period of time and 2- to obtain a number of samples that could reliably inform on the main types of containers producing *Ae. aegypti* [44]. Each container with water was located and recorded from each sampled house and mosquito pupae were taken to the laboratory in 80% ethanol for species identification. The pupal survey methodology used in this study has been described in detail [37].

### Ethics statement

Although the database from which the information was obtained contains identifiable information, only non-identifiable data was provided to the investigators for this work by an individual not otherwise involved in the conduct of the study. Therefore it was determined that this activity did not involve human subjects as described under the human subjects protection regulations at 45 CFR 46.102 (f) and an IRB review was not required.

### Dengue data

Suspected and confirmed cases of dengue by onset of symptoms during the period of study were obtained from the dengue database of the passive surveillance system that is in place for Puerto Rico at the Dengue Branch, Centers for Disease Control and Prevention. Dengue cases reported for the study areas were aggregated and extracted using the boundaries of the census tracts within the GIS. A variable named adjusted dengue cases (ADJDENGUE) was constructed from the accumulated number of dengue cases reported during the second, third and fourth weeks after mosquito sampling. The reason for creating this variable was that recently emerged mosquitoes cannot transmit dengue virus until after a period that includes: biting an infected person and acquiring the virus (2–3 days), incubation of the virus in the vector (7–10 days) [32], then biting a susceptible person. Transmission is followed by the intrinsic incubation of the virus (5 days), and seeing a doctor after symptoms' onset (1–5 days).

The number of dengue cases reported to the passive surveillance system from November 2007 to December 2008 in the study areas in EC and VC were two and six, respectively. This small number of cases did not allow us exploring the associations between mosquitoes, weather variables, and dengue within the investigated areas. One plausible reason for such small number of cases is that the passive surveillance system usually captures a small number of symptomatic cases and misses most asymptomatic ones. Therefore, we decided to explore the relationship between female *Ae. aegypti* abundance in each neighborhood and dengue cases per 100000 inhabitants (inh.) in the San Juan and Carolina municipalities where mosquito surveys were conducted.

### Statistical analyses

Average abundance of female *Ae. aegypti* captured per BG trap per day was compared between neighborhoods using a linear mixed model with ADJRAIN and ADJTEMP as covariates over the 20 sampling dates. A linear mixed model was also used to compare the average number of eggs per ovitrap per sampling period between neighborhoods. A mixed model accounts for the possible temporal correlation in this type of longitudinal data and includes the calculation of random factors for each site. Covariance structure for repeated measures was modeled as being autoregressive of order one. Analyses were performed using SPSS 12.0.

## Results

### Main types of containers producing *Ae. Aegypti*

**Figure 3:**
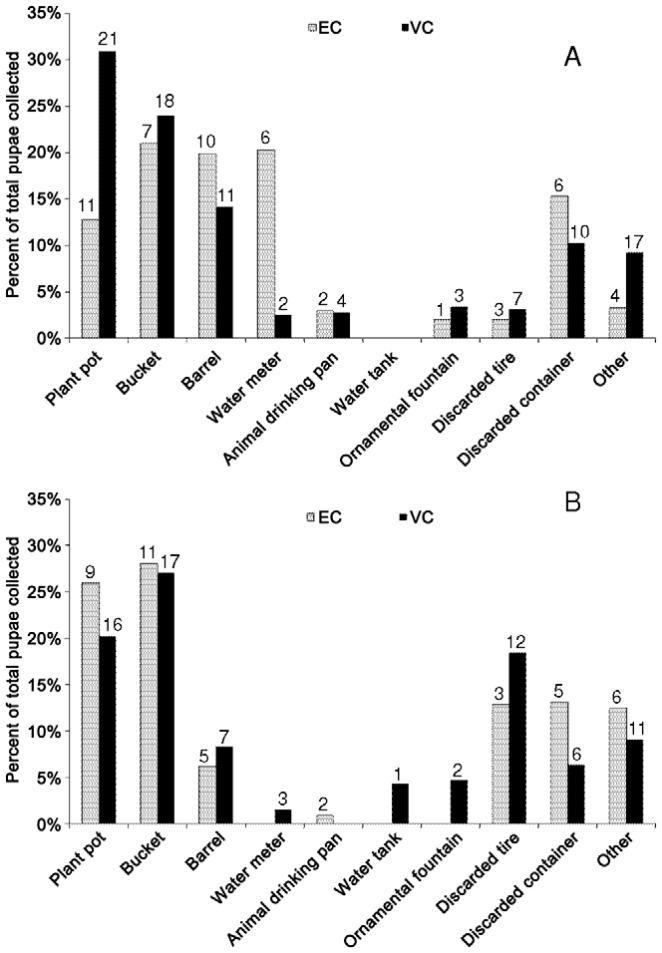
*Aedes aegypti* pupae per type of container. Percentage of all pupae found in pupal surveys conducted during the “drier” (Panel A) and rainy (Panel B) periods, respectively, in neighborhoods “El Comandante” (EC) and “Villa Carolina” (VC), San Juan city, Puerto Rico. Numbers on top of bars indicate how many containers of each type were found with pupae.

During the drier season (January/February 2008), 5-gal buckets, barrels, plant pots, and water meters collectively produced 74% of the *Ae. aegypti* pupae in EC and 72% of the *Ae. aegypti* pupae in VC. Water meters are located in a 1–1.5 gal cavity below ground level in front of every house. During the rainy season, 5-gal buckets, barrels, plant pots, and water meters were producing 60% and 57% of all pupae in EC and VC, respectively. The contribution of barrels and water meters decreased whereas the contribution of tires increased in the rainy season (Figure 3).

Immature *Ae. aegypti* indicators revealed similarities between sites in June 2008: House Index (30% in EC; 32% in VC) and total pupae collected (705 in EC; 746 in VC). The number of pupae per house was not statistically different (log10+1 transformed, t = 0.12; df = 458; P>0.05) between EC (3.13±0.90) and VC (3.17±0.77). By contrast, the mean number of adult *Ae. aegypti* captured per BG trap per day by the end of June 2008 was larger in EC (10.38±1.10) than in VC (6.39±0.44), though the difference was not statistically significant at α = 0.05 (log10+1 transformed, unequal variances assumed, t = 1.79; df = 302; P>0.05). The pupal sex ratios (male: female) were 0.9∶1 in EC and 1∶1 in VC.

#### Adult *Aedes aegypti* dynamics in BG-Sentinel traps

A total of 20 mosquito species was captured in the study sites from November 2007 to December 2008 in BG traps (Table 1). The most abundant mosquito species were *Culex quinquefasciatus* and *Aedes aegypti*. The number of adult *Cx. quinquefasciatus* collected in EC was several times larger than in VC (Table 1). We observed that several houses in the eastern part of EC had septic tanks, but we could not sample them. The number of adult males of *Ae. aegypti* captured in VC was half of that captured in EC (Table 1).

The overall mean numbers of *Ae. aegypti* females and males per trap per day were 4.76±0.22 (±95% CI) and 4.06±0.29, respectively in EC (n = 3059 trap days). There were 3.80±0.14 females and 2.13±0.11 males per trap per day in VC (n = 3048). The results of a linear mixed model comparing the average number of females per trap per day (log10-trasformed) between the two study sites for the 20 temporal observations every three weeks, with ADJRAIN and ADJTEMP as covariates, indicated significant differences between sites (F = 15.9; P<0.01) and significant effects of rainfall (F = 33.2; P<0.01) and temperature (F = 19.3; P<0.01).

**Figure 4:**
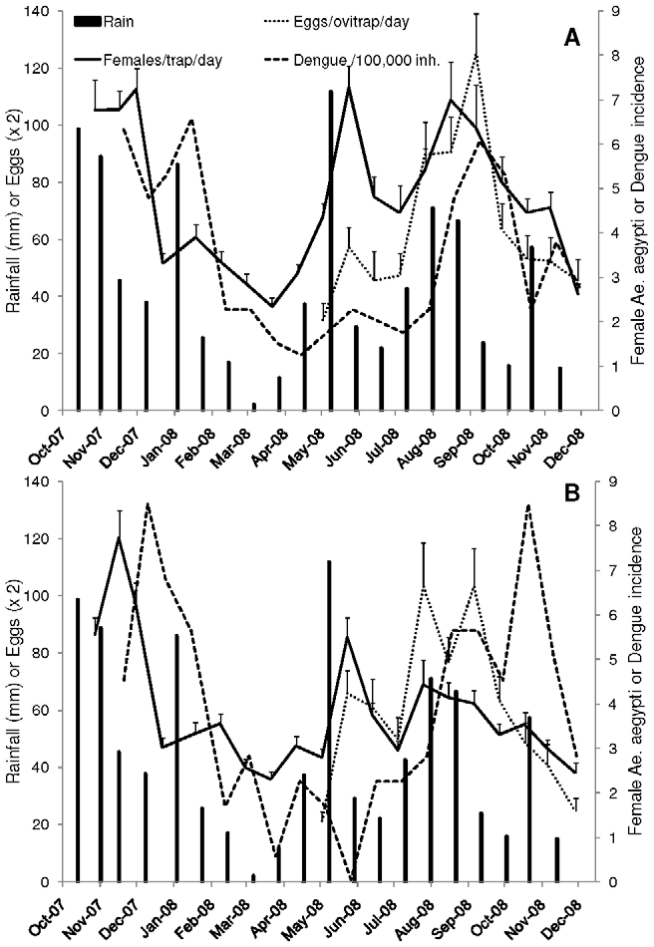
Temporal changes in rainfall, mosquitoes, and dengue. Panel A shows changes in adjusted rainfall (mm), number of *Aedes aegypti* females per BG-Sentinel trap per day, number of eggs per CDC ovitrap per day, and adjusted dengue incidence (cases per 100000 inhabitants) in “El Comandante” (EC) and Panel B shows these parameters in “Villa Carolina” (VC), San Juan city, Puerto Rico from October 2007 to December 2008.

**Figure 5:**
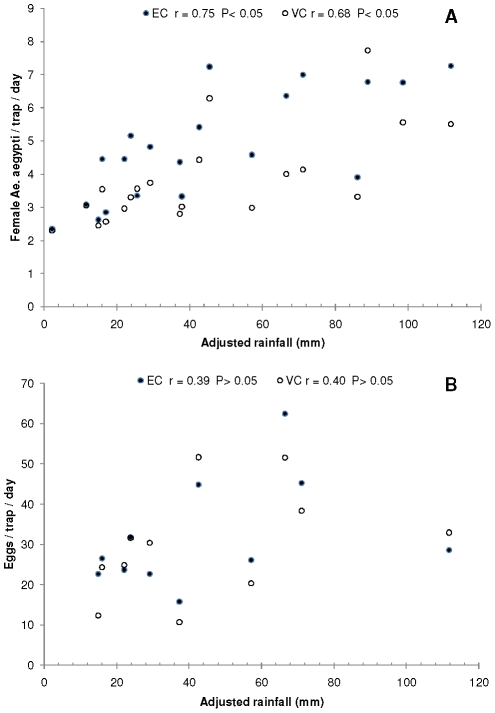
Relationships between mosquitoes and rainfall. Panel A presents the number of female *Ae. aegypti* per BG-Sentinel trap per day versus adjusted rainfall (mm) for each sampling date in “El Comandante” (EC) and “Villa Carolina” (VC), San Juan city, Puerto Rico, and Panel B shows the number of eggs per CDC ovitrap per day versus adjusted rainfall in each neighborhood. The corresponding correlation coefficients and Type I error probabilities are presented next to the location.

The number of female *Ae. aegypti* captured in BG traps decreased from November 2007 through April 2008 in both study areas, concurrently with a sustained decrease in rainfall (Figure 4), with the exception of an intense precipitation event that occurred on January 19–20 that was associated with slight increases in mosquito densities in both neighborhoods (Figure 4). The density of *Ae. aegypti* in both study sites was similar from November 2007 through April 2008, but after that date (rainy season) the density was higher in EC than in VC (Figure 4). Additionally, although rainfall reached minimum values in March - April, mosquito densities did not proportionally decrease in the neighborhoods. Maximum mosquito densities in BG traps during 2008 were registered in June and September in EC and in June and August-September in VC. The number of females *Ae. aegypti* captured in BG traps was positively and significantly correlated with adjusted rainfall (Figure 5A) in both neighborhoods. Adjusted temperature was positively and significantly correlated with the number of *Ae. aegypti* females in BG traps in EC (r = 0.50; P<0.05) but not in VC (r = 0.10; P>0.05).

#### *Aedes aegypti* dynamics in ovitraps

Overall average number of eggs/ovitrap/day was similar in EC (31.94±1.07; n = 1745; Total eggs = 55743) and VC (29.76±1.02; n = 1705; Total eggs = 50737). A linear mixed model did not find significant differences in the average number of eggs per ovitrap between neighborhoods (F = 0.19, P>0.05). The number of eggs in the ovitraps was correlated with the number of females captured per BG trap in EC (r = 0.61; P<0.05) and VC (r = 0.69; <0.05).

The number of mosquito eggs per ovitrap reached peak values in June and September in EC and in June, September and November in VC (Figure 4). The numbers of eggs per ovitrap show a linear tendency with adjusted rainfall for most samples, however the correlation was not significant due to an outlier sample in each neighborhood (Figure 5B). Both outliers corresponded to samples taken in June, right after a large rainfall event that was associated with a peak in the number of females in both neighborhoods (Figure 4). Adjusted temperature was positively correlated with the number of eggs per ovitrap in VC (r = 0.66; P<0.05) but not in EC (r = 0.39; P>0.05).

### Dengue dynamics

**Figure 6:**
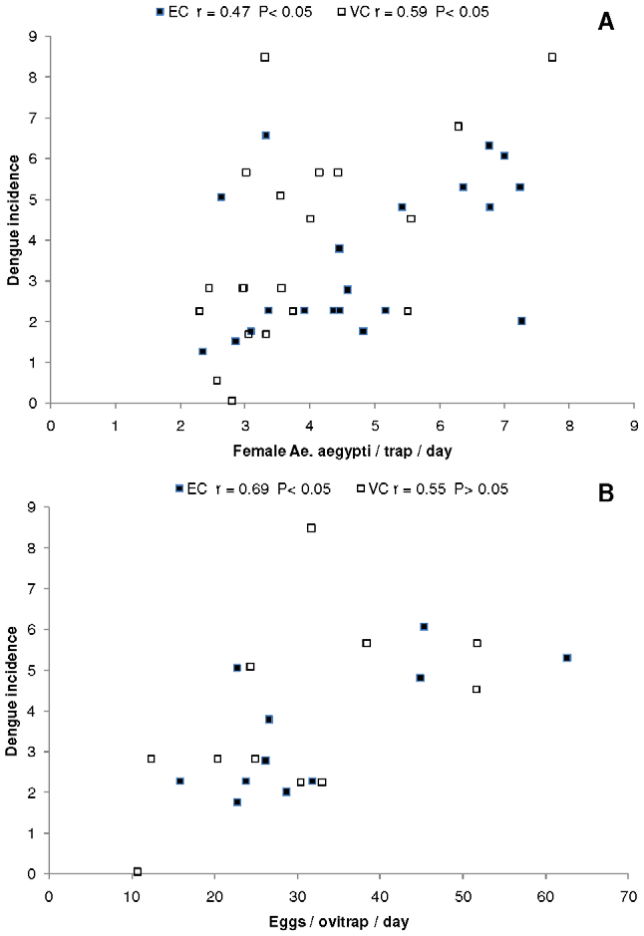
Relationships between dengue incidence and mosquitoes. Panel A presents dengue incidence (cases per 100000 inhabitants) as a function of the number of female *Ae. aegypti* per BG-Sentinel trap per day for each sampling date in “El Comandante” (EC) and “Villa Carolina” (VC), San Juan city, Puerto Rico, and Panel B shows dengue incidence as a function of the number of eggs per CDC ovitrap per day in each neighborhood. The corresponding correlation coefficients and Type I error probabilities are presented next to the location.

Dengue prevalence during the period of study was similar in San Juan (71 cases per 100000 inh.) and Carolina (77 cases per 100000 inh.) municipalities. Dengue incidence reached maximum values in San Juan municipality during the beginning of 2008 and in September 2008; in both cases, peak dengue incidence followed peaks in female mosquito density in EC (Figure 4A). The peaks of mosquito density observed in June 2008 were not associated with a large increase in dengue incidence (Figure 4). There were also two maxima of dengue incidence in Villa Carolina municipality; one at the end of December 2007 and the other in November 2008 (Figure 4B). Dengue incidence reached the lowest levels by the end of the “drier season” (San Juan) or beginning of the rainy season (Carolina; Figure 2, 4). Dengue incidence was positively and significantly correlated with the number of female *Ae. aegypti* in both neighborhoods (Figure 6A). Similarly, dengue incidence was positively correlated with the number of eggs per ovitrap in EC but did not reach statistical significance in VC (Figure 6B). It can be noted that mosquito density never reached values below two in BG traps or below ten in ovitraps during the study (Figure 6). With the exception of one sample in VC (May 2008), all vector density values observed in this study were associated with dengue incidence.

## Discussion

### *Ae. aegypti* dynamics and dengue endemism

The pupal surveys conducted during the drier and wetter parts of the year in San Juan city during the end of 2007 and 2008 revealed that most pupae were produced in containers whose water content was managed by humans: water storage vessels (5-gal pails, barrels), leaking-water meters, and plant pots. This is the first report on water meters as productive aquatic habitats for *Ae. aegypti*. Containers that were mainly filled with water by rains increased during the rainy season, most notably used tires (Figure 3). Therefore, it would seem that the reason why the *Ae. aegypti* population did not reach very low levels at a time when rainfall was scant (Figure 4) was due to the production of mosquitoes in containers managed by people. This phenomenon was remarkably similar in both neighborhoods. A longitudinal study that investigated the temporal changes in aquatic habitats and oviposition of *Ae. aegypti* in northern Venezuela showed similar dynamics, although in such study the main habitats producing mosquitoes during the prolonged dry seasons were 55-gal barrels used for water storage [14]. Lambdin et al. [45] found that buckets, barrels, and tires were the most productive containers during the dry and wet seasons in American Samoa. It is perplexing that *Ae. aegypti* mosquitoes were being produced in water storage containers and tires in Puerto Rico because the country has a reliable supply of tap water and a tire-recycling program. Our results seem to add support to the hypothesis that dengue endemicity (uninterrupted transmission) is favored by the persistent production of mosquitoes in containers whose water is managed by people.

### Weather effects

The present longitudinal study of *Aedes aegypti* in neighborhoods of San Juan City revealed significant changes in the number of adult mosquitoes in BG traps that were positively associated with rainfall and temperature (Figures 4, 5). The typical bimodal rainfall pattern of northern Puerto Rico [46] was associated with two main peaks in mosquito abundance, particularly in EC (Figure 4). The first peak in mosquito density was associated with a small hump in the figure of dengue cases in June and the second peak in mosquito density preceded maximum dengue incidence later in the year (Figure 4). It could be observed that dengue incidence decreased after reaching its peak, in association with concomitant reductions in rainfall and *Ae. aegypti* density (Figure 4). Thus, the present study is in agreement with a previous longitudinal study of *Ae. aegypti* in Puerto Rico that showed significant effects of rainfall on larval indices and dengue incidence [36]. These authors suggested that such a relationship was patent in the drier cities of the island because rainfall is more seasonal. The lack of significant effects of weather on adult *Ae. aegypti* in a previous study in San Juan city in northern Puerto Rico, where rainfall is more uniformly distributed throughout the year [38], seemed to confirm such hypothesis. However, in the present study *Ae. aegypti* dynamics was strongly driven by weather and human activity. The only previous study of *Ae. aegypti* dynamics using BG traps in relation to weather variables was done in tropical Cairns, Australia [47]. The authors used weekly values of temperature, rainfall, and relative humidity and explored varying time lag effects using multiple regression analyses. They did not find significant effects of rainfall at any time lags, but significant effects of relative humidity with a lag of two weeks and mean daytime temperature at lag 0.

Oviposition (eggs/ovitrap/day) was also influenced by rainfall and this variable was correlated with the abundance of *Ae. aegypti* females in BG traps. Both female adults and oviposition were correlated with dengue incidence. Such an association could be more easily seen in EC (Figures 4, 6). Mogi et al. [48] reported marked seasonal changes in *Ae. aegypti* oviposition associated with the rainy season, with maximum numbers occurring one month before the peak of dengue cases in northern Thailand. It would appear that ovitraps could be used as inexpensive indicators of the risk of dengue transmission, although perhaps these traps may not consistently reflect overall mosquito population abundance. BG traps capture *Ae. aegypti* in various physiological states but underestimate some groups, such as nulliparous females [49], whereas ovitraps reflect the number of ovipositing or gravid females [50], [51]. Ovitraps have been used to monitor temporal changes in *Ae. aegypti* populations and to assess the impact of insecticide treatments [52]. The use of ovitraps for surveillance purposes can be made easier because the number of eggs per ovitrap can be significantly related to the percentage of positive traps using an empirical model, so once the model is developed there is no need to count the eggs collected in the traps [53]. We propose a closer examination of the value of standard CDC ovitraps or similar devices that monitor gravid females as indicators of dengue transmission. The results obtained in this investigation suggest that the levels of *Ae. aegypti* females per BG trap or the number of eggs per ovitrap should be reduced well below two and ten, respectively to prevent dengue transmission (Figure 6). Mogi et al. [48] did not observe dengue hemorrhagic fever cases in Chiang Mai, Thailand when the number of eggs in ovitraps baited with water was two or less.

### Variation between neighborhoods

Pupal surveys indicated rather similar entomological indices and numbers of pupae of *Ae. aegypti* in both study areas in June, 2008. However, the number of adult *Ae. aegypti* captured in BG traps was significantly greater in EC than in VC. Among the main differences observed between the two areas was the fact that there was a deficit of male *Ae. aegypti* mosquitoes captured in BG traps in VC (Table 1). This unbalance in the proportion of males was not observed in the pupal surveys, suggesting that it resulted later on during the adult life of the mosquitoes. Additionally, an outstanding difference in dynamics between the two sites was observed in August and September, when it appeared like the second peak in mosquito abundance in VC was trimmed in comparison with EC (Figure 4). A hypothesis explaining this difference between neighborhoods is that there was more frequent application of truck-mounted, Ultra Low Volume (ULV) spraying of insecticide in VC, particularly during the dengue season. Unfortunately, we could not gather enough data to document when and where insecticides were applied in each neighborhood. Yet, of the 78 municipalities of Puerto Rico, Carolina municipality was the one that spent the most in the chemical control of mosquitoes [54]. Focks et al. [55] reported that truck-mounted ULV spraying killed an average of 88% of the males and 30% of the females in New Orleans, Louisiana. This greater impact of insecticide spraying on *Ae. aegypti* males is consistent with our observations in VC. However, if insecticide spraying actually reduced the number of mosquitoes captured in BG traps, it did not significantly affect the number of eggs in ovitraps since there were no differences between neighborhoods. The Carolina municipality also carries out insecticide spraying to eliminate other biting mosquito species that come from nearby marshes and mangroves (Table 1). An additional difference between neighborhoods was the comparatively larger density of *Culex quinquefasciatus* observed in EC (Table 1), which was probably due to the presence of septic tanks that were observed in some of the less urbanized sectors of EC. It would appear that current levels of mosquito population control were insufficient to make a difference in terms of dengue transmission between municipalities because dengue prevalence during the study was similar in both administrative areas.

## References

[1] Johansson MA, Cummings DAT, Glass GE (2009). Multiyear climate variability and dengue—El Niño Southern Oscillation, weather, and dengue incidence in Puerto Rico, Mexico, and Thailand: A longitudinal data analysis.. PLoS Med.

[2] Tipayamongkholgul M, Fang CT, Klinchan S, Lui CM, King CC (2009). Effects of the El Niño-Southern Oscillation on dengue epidemics in Thailand, 1996–2005.. BMC Public Health.

[3] Rigau-Pérez JG, Clark GG, Gubler DJ, Reiter P, Sanders EJ (1998). Dengue and dengue haemorrhagic fever.. Lancet.

[4] Ooi EE, Gubler DJ (2009). Global spread of epidemic dengue: the influence of environmental change.. Future Virol.

[5] Hales S, Weinstein P, Souares Y, Woodward A (1999). El Niño and the dynamics of disease transmission.. Environ Health Persp.

[6] Amarakoon D, Chen A, Rawlins S, Chadee D, Taylor M (2008). Dengue epidemics in the Caribbean-temperature indices to gauge the potential for onset of dengue.. Mitig Adapt Strat Glob Change.

[7] De Souza SS, da Silva IG, da Silva HHG (2010). Associação entre incidência de dengue, pluviosidade e densidade larvária de *Aedes aegypti* no Estado de Goiás.. Rev Soc Bras Med Trop.

[8] Chadee D, Shivnauth B, Rawlins SC, Chen AA (2007). Climate, mosquito indices and the epidemiology of dengue fever in,Trinidad (2002–2004).. Ann Trop Med Parasitol.

[9] Kuno G, Gubler DJ, Kuno G (1997). Factors influencing the transmission of dengue viruses.. Dengue and dengue hemorrhagic fever.

[10] Hay S, Myers MF, Burke DS, Vaughn DW, Endy T (2000). Etiology of interepidemic periods of mosquito-borne disease.. PNAS.

[11] Wearing HJ, Rohani P (2006). Ecological and immunological determinants of dengue epidemics.. Proc Natl Acad Sci USA.

[12] Cazelles B, Chavez M, McMichael AJ, Hales S (2005). Nonstationary influence of El Niño on the synchronous dengue epidemics in Thailand.. Plos Med.

[13] Jury MR (2008). Climate influence on dengue epidemics in Puerto Rico.. Int J Environ Health Res.

[14] Barrera R, Avila JL, Navarro JC (1996). Dinámica poblacional de *Aedes aegypti* (L.) en centros urbanos con deficiencia en el suministro de agua.. Acta Biol Venez.

[15] Cummings DAT, Irizarry RA, Huang NE, Endy TP, Nisalak A (2004). Travelling waves in the occurrence of dengue haemorrhagic fever in Thailand.. Nature.

[16] McElroy KL, Santiago GA, Lennon NJ, Birren BW, Henn MR (2011). Endurance, refuge, and reemergence of dengue virus type 2, Puerto Rico, 1986 2007.. Emerg Infect Dis.

[17] Adams B, Boots M (2010). How important is vertical transmission in mosquitoes for the persistence of dengue? Insights from a mathematical model.. Epidemics.

[18] Eamkan P, Nisalak A, Foy HM, Chareonsook OA (1989). Epidemiology and control of dengue virus infections in Thai villages in 1987.. Am J Trop Med Hyg.

[19] Gonzalez R, Suarez MF (1995). Sewers: The principal *Aedes aegypti* breeding sites in Cali, Colombia.. Am J Trop Med Hyg.

[20] Kay BH, Ryan PA, Russell MB, Holt JS, Lyons SA (2000). The importance of subterranean mosquito habitat to arbovirus vector control strategies in north Queensland, Australia.. J Med Entomol.

[21] Montgomery BL, Ritchie SA (2002). Roof gutters: A key container for *Aedes aegypti* and *Ochlerotatus notoscriptus* (Diptera: Culicidae) in Australia.. Am J Trop Med Hyg.

[22] Barrera R, Amador M, Diaz A, Smith J, Munoz-Jordan JL, Rosario Y (2008). Unusual productivity of *Aedes aegypti* in septic tanks and its implications for dengue control.. Med Vet Entomol.

[23] Russell BM, McBride JH, Mullner H, Kay BH (2002). Epidemiological significance of subterranean *Aedes aegypti* breeding sites to dengue virus infection in Charters Towers 1993.. J Med Entomol.

[24] MacKay AJ, Amador M, Diaz A, Smith J, Barrera R (2009). Dynamics of *Aedes aegypti* and *Culex quinquefasciatus* in septic tanks.. J Am Mosq Control Assoc.

[25] Foo LC, Kim TW, Lee HL, Fang R (1985). Rainfall, abundance of *Aedes aegypti* and dengue infection in Selangor, Malaysia.. SE Asian J Trop Med Public Health.

[26] Schultz GW (1993). Seasonal abundance of dengue vectors in Manila, Republic of the Philippines, SE Asian.. J Trop Med Public Health.

[27] Neto VSG, Rebelo JMM (2004). Aspectos epidemiológicos do dengue no Municipio de São Luís, Maranhão, Brasil 1977–2002.. Cad Saude Publica.

[28] Dibo MR, Chierotti AP, Ferrari MS, Mendonça AL, Neto FC (2008). Study of the relationship between *Aedes aegypti* egg and adult densities, dengue fever and climate in Mirassol, state of São Paulo, Brazil.. Mem Inst Oswaldo Cruz.

[29] Barbosa GL, Lorenço RW (2010). Análise da distribuição espaço-temporal de dengue e da infestação larvária no município de Tupã, Estado de São Paulo.. Rev Soc Bras Med Trop.

[30] Sheppard PM, MacDonald WW, Tonn RJ, Grab B (1969). The dynamics of an adult population of Aedes aegypti in relation to dengue haemorrhagic fever in Bangkok.. J Anim Ecol.

[31] Tonn RJ, Sheppard PM, MacDonald WW, Bang YH (1969). Replicate surveys of larval habitats of *Aedes aegypti* in relation to dengue haemorrhagic fever in Bangkok, Thailand.. Bull Wld Hlth Org.

[32] Watts DM, Burke DS, Harrison BA, Whitmire RE, Nisalak A (1987). Effect of temperature on the vector efficiency of *Aedes aegypti* for dengue 2 virus.. Am J Trop Med Hyg.

[33] Focks DA, Barrera R (2006). Dengue transmission dynamics: assessment and implications for control.. Report of the Scientific Working Group Meeting on Dengue.

[34] Halstead SB (2008). Dengue virus-mosquito interactions.. Annu Rev Entomol.

[35] Gould DJ, Mount GA, Scanlon JE, Ford HR, Sullivan MF (1970). Ecology and control of dengue vectors on an island in the Gulf of Thailand.. J Med Entomol.

[36] Moore CG, Cline BL, Ruiz-Tiben E, Lee D, Romney-Joseph H (1978). *Aedes aegypti* in Puerto Rico: Environmental determinants of larval abundance and relation to dengue virus transmission.. Am J Trop Med Hyg.

[37] Barrera R, Amador M, Clark GG (2006). Use of the pupal survey technique for measuring *Aedes aegypti* (Diptera: Culicidae) productivity in Puerto Rico.. Am J Trop Med Hyg.

[38] Scott TW, Morrison AC, Lorenz LH, Clark GG, Strickman D (2000). Longitudinal studies of *Aedes aegypti* (Diptera: Culicidae) in Thailand and Puerto Rico: Population dynamics.. J Med Entomol.

[39] Johansson MA, Dominici F, Glass GE (2009). Local and global effects of climate on dengue transmission in Puerto Rico.. PLoS NTD.

[40] Barrera R (2010). Dinámica del dengue y *Aedes aegypti* en Puerto Rico.. Rev Biomed.

[41] Fortin MJ, Dale M (2008). Spatial analysis: A guide for ecologists.

[42] Reiter P, Amador MA, Colon N (1991). Enhancement of the CDC ovitrap with hay infusions for daily monitoring of *Aedes aegypti* populations.. J Am Mosq Control Assoc.

[43] Smith J, Amador M, Barrera R (2009). Seasonal and habitat effects on dengue and West Nile virus vectors in San Juan, Puerto Rico.. J Am Mosq Control Assoc.

[44] Barrera R, Amador M, Clark GG (2006). Sample-size requirements for developing targeted control strategies for dengue using the pupal/demographic survey.. Ann Trop Med Parasitol.

[45] Lambdin BH, Schmaedick MA, McClintock S, Roberts J (2009). Dry season production of filariasis and dengue vectors in American Samoa and comparison with wet season production.. Am J Trop Med Hyg.

[46] Jury MR, Malmgren BA, Winter A (2007). Subregional precipitation climate of the Caribbean and relationships with ENSO and NAO.. J Geophys Res.

[47] Azil AH, Long SA, Ritchie SA, Williams CR (2010). The development of predictive tools for pre-emptive dengue vector control: a study of *Aedes aegypti* abundance and meteorological variables in north Queensland, Australia.. Trop Med Int Health.

[48] Mogi M, Khamboonruang C, Choochote W (1988). Ovitrap surveys of dengue vector mosquitoes in Chiang Mai, northern Thailand: seasonal shits in relative abundance of *Aedes albopictus* and *Ae. aegypti*.. Med Vet Entomol.

[49] Ball TS, Ritchie SR (2010). Sampling biases of the BG-Sentinel trap with respect to physiology, age, and body size of adult *Aedes aegypti*.. J Med Entomol.

[50] Facchinelli L, Valerio L, Pombi M, Reiter P, Costantini C (2007). Development of a novel sticky trap for container breeding mosquitoes and evaluation of its sampling properties to monitor urban populations of *Aedes albopictus*.. Med Vet Entomol.

[51] Honorio NA, Codeço CT, Alves FC, Magalhães MAFM, Lourenço de Oliveira R (2009). Temporal distribution of *Aedes aegypti* in different districts of Rio de Janeiro, Brazil, measured by two types of traps.. J Med Entomol.

[52] Focks DA (2003). A review of entomological sampling methods and indicators for dengue vectors.

[53] Mogi M, Choochote W, Khamboonruang C, Suwanpanit P (1990). Applicability of presence-absence and sequential sampling for ovitrap surveillance of *Aedes* (Diptera Culicidae) in Chiang Mai, Northern Thailand.. J Med Entomol.

[54] Pérez-Guerra CL, Halasa YA, Rivera R, Peña M, Ramírez V (2011). Economic cost of dengue public prevention activities in Puerto Rico.. Dengue Bull.

[55] Focks DA, Kloter KO, Carmichael GT (1987). The impact of sequential ultra low volume ground aerosol applications of malathion on the population dynamics of *Aedes aegypti*.. Am J Trop Med Hyg.

